# Evaluation of a Pregnancy Options Counseling Curriculum for Pediatric Residents

**DOI:** 10.1016/j.jadohealth.2024.11.003

**Published:** 2024-12-15

**Authors:** Laura Kirkpatrick, Lauren A. Bell, Theresa Borcky, Hannah Boutros-Khoury, Jayde Hooven-Davis, Jacquelin Rankine, Cynthia Robbins, Tahniat Syed, Nicholas Szoko, Bianca A. Allison

**Affiliations:** a Department of Pediatrics, University of Pittsburgh School of Medicine, Pittsburgh, Pennsylvania; b UPMC Children’s Hospital of Pittsburgh, Pittsburgh, Pennsylvania; c Department of Preventive Medicine, The University of Tennessee Health Science Center, Memphis, Tennessee; d Department of Pediatrics, University of North Carolina School of Medicine, Chapel Hill, North Carolina; e Pediatrics, Maine Health, Portland, Maine

**Keywords:** Pregnancy options counseling, Pediatric residency, Resident curriculum, Adolescent health, Sexual and reproductive health

## Abstract

**Purpose:**

To evaluate an adolescent-centered pregnancy options counseling curriculum for pediatric residents.

**Methods:**

In 2022, we assessed the curriculum in one residency by randomizing half the residents to a pretraining or posttraining Observed Structured Clinical Exam (OSCE). We scored the OSCE by 15-item rubric and analyzed results by Wilcoxon Rank-Sum tests. From 2022 to 2023, we implemented the curriculum across 3 residencies. Surveys assessed knowledge and self-rated abilities before, immediately after, and 6-month posttraining. Two residencies also surveyed controls. We analyzed results using mixed-effects and ordinal logistic regression.

**Results:**

2022: 25 residents participated in the OSCE (13 pregroup, 12 post-group). Median score increased from 9/15 (interquartile range 8/15, 10/15) pretraining to 13/15 (13/15, 14/15) posttraining (*p* < .05).

2022–2023: Residents in the training arm completed presurveys (n = 79), immediate postsurveys (n = 65), and 6-month postsurveys (n = 40). Median baseline knowledge score was 57% (24%, 83%), which improved immediately posttraining [90% (81%, 95%), *p* < .05] and was sustained at 6 months [86% (57%, 90%), *p* < .05]. Median baseline self-rated ability to discuss pregnancy options was 8/12 (7/12, 9/12) and increased immediately posttraining [12/12 (10/12, 12/12), *p* < .05] with sustained improvement at 6 months [9/12 (8/12, 11/12), *p* < .05]. Median baseline self-rated ability to provide pregnancy-related referrals was 6/12 (5/12, 8/12) which improved posttraining [10/12 (9/12, 12/12), *p* < .05)] with sustained improvement at 6 months [8/12 (6/12, 9/12), *p* < .05]. Control residents completed presurveys (n = 140) and 6month postsurveys (n = 94). Trained residents performed better at 6 months on all measures (*p* < .05).

**Discussion:**

Pregnancy options counseling training for pediatrics residents led to improved knowledge, abilities, and behavior.

Though down-trending, adolescent pregnancy remains common in the United States compared to other high-income countries with 15.4 births per 1000 females aged 15–19 years in 2020. [1–4] The American Academy of Pediatrics (AAP) recommends that pediatricians provide unbiased, comprehensive pregnancy options counseling (POC) to pregnant adolescents, including discussion of abortion, adoption, and parenting. [5] However, our prior qualitative research revealed that adolescents who are pregnant often receive no or poor-quality POC in pediatric settings and at times have interactions with health professionals that can be highly upsetting at a potentially pivotal time in their lives. [6].

Pediatricians must be prepared to encounter pregnant patients and deliver high-quality POC as recommended by the AAP, yet there is a gap in the quality of care. One potential solution is to incorporate POC training into pediatrics residency. However, training modalities specific to pediatric residents are lacking in the literature. [7–10] Curricula specific to pediatric residents are needed because pregnancy care is less commonly encountered clinically in pediatric training compared with other disciplines such as Family Medicine or Obstetrics-Gynecology. In addition, pregnant adolescents may have different developmental needs and face different medico-legal barriers related to accessing pregnancy options than adults, necessitating an adolescent-centered curriculum for pediatricians. [3,11].

The development of the curriculum is described elsewhere. [12,13] Briefly, in 2019, we developed and delivered a 2-hour curriculum on POC to all Post Graduate Year (PGY)-1 pediatric residents at a single pediatric residency program. [12] The original curriculum included a brief didactic followed by simulated cases. As previously published, [12] residents showed significant improvement in knowledge and self-rated ability in POC on immediate and 6-month posttraining survey assessments compared to preassessments. Residents also showed better performance on these assessments than untrained senior (PGY-2 and PGY-3) pediatric residents, suggesting that the training adds novel skill development to residency. The curriculum was revised from 2020 to 2022 to incorporate feedback from people who were pregnant during adolescence and community professionals serving pregnant adolescents. [6,13] The most significant change to the curriculum was the addition of a values clarification exercise.

Given these promising pilot results, we first evaluated the revised curriculum with a pretraining/posttraining Observed Structured Clinical Exam (OSCE) at the original site. Next, we conducted a quasiexperimental pilot study implementing the curriculum at 2 additional pediatric residency programs, along with the original site, to assess its effect on pediatric residents’ knowledge and self-rated abilities pre/post and by comparison over time with untrained controls.

## Methods

### Curriculum

The curriculum implemented at multiple sites consisted of a brief didactic, values clarification exercise, and small group role-play sessions with a facilitator and an actor portraying a pregnant adolescent. We included up to 5 role-play scenarios, with each participant given 5–7 minutes to act in a clinician role and provide POC for the portrayed adolescent experiencing pregnancy. We designed an optional sixth scenario for individuals with conscientious objections to providing comprehensive POC, in which they could role-play transitioning care to another clinician for comprehensive POC, as recommended in the AAP guidelines for individuals unable to provide nonjudgmental, factual POC. [5] We designed the curriculum to last approximately 2 hours.

Adolescent-centered features of the curriculum include the values clarification exercise and training on developmentally-tailored suggested language, privacy and confidentiality, troubleshooting challenges adolescents may face in accessing care independently or in seeking help from supportive adults, and pertinent laws affecting adolescents such as parental consent laws for abortion. At their discretion, sites provided resources for further learning about relevant laws and policies, such as the Guttmacher Institute, Repro Legal Helpline and If/When/How. A key feature of our curriculum that distinguishes it from prior educational initiatives is its emphasis on role-plays, which allow for experiential learning through skills practice with formative feedback. We have included the facilitation guide for the curriculum as Appendix 1.

We also modified the curriculum for performance in a state with threatened limitations to abortion access following the 2022 *Dobbs* versus *Jackson Women*’*s Health Organization* US Supreme Court Decision (Site #3). The modified curriculum included supplemental information, such as resources in adjacent states where abortion was accessible, and harm reduction resources for patients who express that they are planning self-managed abortion.

### Single-Site OSCE

#### Participants.

We first implemented the curriculum at the original site’s pediatric residency program in Spring 2022, delivering it to all PGY-1 residents. We evaluated it through pre-OSCE/post-OSCE assessments. Residents were randomly assigned to complete the OSCE either before or immediately after the 2-hour POC curriculum using 1:1 block randomization. Due to limitations related to scheduling, standardized patients were not blinded to whether participants were in the pre-OSCE or post-OSCE group.

#### OSCE development.

We developed an OSCE in which a standardized patient would portray a pregnant adolescent receiving an initial diagnosis of pregnancy in the pediatric emergency department. We aimed for the scenario to be similar to the cases included in the curriculum role-plays, without copying any particular case. The scenario was designed to last a maximum of 10 minutes. OSCE materials included detailed scenario background for the standardized patient with sample dialogue and a more limited information sheet introducing the scenario for the resident. Standardized patients participated in a training and practice session prior to the OSCE. Content experts in adolescent medicine and pediatrics reviewed and approved OSCE materials.

#### Data collection.

We developed a novel 15-item rubric for the standardized patient to grade the residents’ performance. The rubric addressed the following 5 domains: (1) disclosing pregnancy test results; (2) discussing pregnancy options; (3) ensuring support and safety; (4) planning next steps; and (5) overall communication skills. The rubric was assessed for face and content validity by content experts in adolescent medicine and pediatrics.

Each item on the rubric received a single binary score (i.e., 0 or 1) regarding whether a specific behavior was present. The item scores were added for the domain subscore (range 0–3 for all domains except discussing pregnancy options which was scored with range 0–4). Domain subscores were added for the full performance score (range 0–15). The standardized patient training session included practice using the 15-item rubric. OSCE materials, including standardized patient background, information sheet for residents, and 15-item scoring rubric are included as Appendix 2. Prior to the OSCE, participants completed a brief demographic survey. Following the OSCE, the standardized patient provided them with feedback.

#### Data analysis.

We summarized demographic data using descriptive statistics and compared performance scores for pre-OSCE and post-OSCE groups using Wilcoxon Rank-Sum tests. The University of Pittsburgh Institutional Review Board deemed this study exempt.

### Multisite Evaluation

#### Implementation across sites.

We implemented the curriculum at 2 additional sites beginning in Fall 2022 along with the original site in Spring 2023. The original site (Site #1) implemented the curriculum for all PGY-1 residents. The residency class was divided into 4 groups of 8–10 residents, with a coverage schedule. Each group completed the training in a dedicated 2-hour time block created by the residency program. The didactic was delivered to all residents by one facilitator, and then residents were divided into small groups of 4–5 residents to complete the role-plays with one to 2 facilitators and one actor per group.

The first additional site (Site #2) implemented the curriculum in 2 consecutive one-hour blocks for PGY-1 residents during existing protected educational time (i.e., noon conference). The first session consisted of the didactic plus 2 role-play scenarios. The second session consisted of 3 additional role-play scenarios plus a wrap-up. Two facilitators led the didactic in the first session. For role-plays, the PGY-1s divided into 3 small groups of 3–4 residents, each with a facilitator. Three actors rotated among the 3 groups to perform the cases.

The second additional site (Site #3) implemented the curriculum in a 2-hour block of protected educational time during the Adolescent Medicine rotation for all participating pediatrics and medicine-pediatrics residents (PGY-2-PGY-4) and some fellows in Adolescent Medicine (PGY-4-PGY-7). Three to six learners attended each session. The didactic was led by a facilitator, and then the full group engaged in role-plays with one facilitator and one actor.

Each site adopted mechanisms for sharing resources with the residents and patients. Each training session included distribution of a handout where the first page consisted of suggested language for POC and subsequent pages included appropriate local and national resources and referrals to provide patients. Site #1 also created discharge education in the electronic medical record for pregnant patients about different pregnancy options and resources. Sites #2 and #3 similarly created electronic medical record smart phrases addressing similar content though with resources tailored to their local areas.

#### Data collection.

Residents participating in the training completed evaluation surveys immediately before, immediately after, and at a minimum of 6 months after training. We had previously developed the survey instrument for a pilot evaluation of the curriculum, the development and validity testing of which is described elsewhere. [12] In addition, at Site #2 and Site #3, untrained residents also completed surveys at baseline and 6 months to serve as a control group in a quasiexperimental study. We could not recruit controls at Site #1 because all pediatrics residents at this site had been trained in either a previous or current version of this curriculum over the past 5 years.

Each of the surveys (i.e., before, immediately after, and 6 months after) included 3 multiple-choice test questions assessing knowledge of pregnancy options, local parental consent laws for abortion, and local pregnancy-related resources. The results of the test questions were aggregated into a single knowledge score reflecting percent correct across the 3 questions (range 0%–100%). The survey also included Likert-type questions (1-worst rating, 4-best rating) for residents to self-rate in 3 following domains: (1) knowledge of local pregnancy resources (1 question); (2) ability to discuss parenting, abortion, and adoption (3 separate questions); and (3) ability to provide referrals for prenatal care, abortion, and adoption (3 separate questions). Knowledge of pregnancy resources was scored independently (range 1–4). The second 2 domains were subscored separately for parenting, abortion, or adoption (range 1–4 for each), and then each domain was summed to create an aggregated score for discussing pregnancy options (range 3–12) or aggregated score for discussing referrals (range 3–12).

The presurvey also included demographics questions. The immediate postsurvey included Likert-type questions to evaluate the perceived importance, value, and usefulness of the curriculum (1-worst rating, 4-best rating). Residents’ survey responses across time points were linked using a unique code. The survey instrument for Site #1 is included as Appendix 3. The survey was structured identically across sites, though exact answer choices changed depending on the residency program’s local laws and resources (i.e., knowledge question about parental consent laws or local abortion and pregnancy-related resources).

#### Data analysis.

We summarized demographic data and ratings for importance, value, and usefulness using descriptive statistics. We conducted 2 following sets of longitudinal analyses: (1) comparisons across time points for residents in the training arm to evaluate for change over time compared to baseline and (2) comparisons across time points between the training arm and the control arm using an interaction term between group assignment and time-point to assess for differential effect over time.

We analyzed continuous data for knowledge score across time points using a mixed-effects regression model with fixed-effects for site, baseline reported prior training in POC and baseline PGY year, and a random intercept for participant. We treated Likert-type data as ordinal and analyzed it using a mixed-effects ordinal logistic regression model with fixed-effects for site, baseline reported prior training in POC and baseline PGY year, and a random intercept for participant.

We conducted all statistical analyses in STATA (Version 18; College Station, TX). For all analyses, the alpha level was *p* < .05. The University of Pittsburgh Institutional Review Board, the University of North Carolina Institutional Review Board, and the University of Indiana Institutional Review Board deemed this study exempt.

## Results

### Single-site OSCE

25 PGY-1 residents participated in the OSCE (13 pregroup, 12 postgroup) at Site #1 (Table 1). The pre-OSCE group had a median age of 27 years and was 77% female (n = 10) with 15% (n = 2) having received prior training in POC. The post-OSCE group had a median age of 27 years and was 50% female (n = 6) with no participants having received prior training in POC.

Median pretraining performance score was 9/15 (interquartile range 8/15, 10/15) and median posttraining performance score was 13/15 (13/15, 14/15) (*p* < .05) (Table 2). There were significant (*p* < .05) pre/post differences on all domain subscores (i.e. disclosing pregnancy test results, discussing options, ensuring supports and safety, planning next steps) except for overall communication skills, which were similarly high.

### Multisite Evaluation

#### Participants.

A total of 79 residents and fellows participated in the curriculum. 79 of these residents completed presurveys, 65 completed immediate postsurveys, and 40 completed 6-month surveys across 3 sites. The median age of those completing presurveys was 28 years and this group was 71% female (n = 56) with 16% (n = 13) having received prior training in POC. The median age of those completing immediate postsurveys was 28 years with 69% of this group being female (n = 44) and 15% (n = 10) having received prior training in POC. The median age of those completing 6-month surveys was 28 years, with the group being 69% female (n = 27) and with 23% (n = 9) of this group having received prior training in POC.

140 residents and fellows, who did not participate in the curriculum, completed baseline surveys and 94 completed 6-month surveys to serve as a control group. The median age of those completing the presurveys was 29 years and this group was 81% female (n = 114) with 16% (n = 23) having completed prior POC training. The median age of those completing 6-month surveys was 29 years, and this group was 73% female (n = 69) with 18% (n = 17) having completed prior training in POC. Further demographics for training and control group participants are detailed in Table 3.

#### Preanalysis/postanalysis for training arm.

Knowledge scores were significantly higher on immediate and 6-month postsurveys relative to baseline (*p* < .05). Compared to baseline, there were significantly increased odds of higher self-rated knowledge, discussion, and referral scores and all sub-scores on immediate and 6-month postsurveys (*p* < .05) (Tables 4 and 5).

#### Comparative analysis of training arm versus control arm.

The training arm participants outperformed control arm participants on all measures at 6 months. An analytic variable evaluating for an interaction between time and training was statistically significant (*p* < .05) for all metrics, indicating a statistical difference in the performance of these groups on all metrics over time. Odds ratios for these analyses are detailed in Supplementary Table 6.

#### Resident evaluation of curriculum quality.

On the immediate postsurvey, residents rated (1) the importance of the training; (2) the value of the training to their education; and (3) the usefulness of the training all a median of 4/4 (interquartile range 4/4, 4/4).

#### Opt-out.

No residents opted out of the training at any of the 3 sites, though one resident at Site #1 chose to participate in the conscientious objection role-play option. The low opt-out rate supports the acceptability of this training in pediatric residencies.

## Discussion

We implemented a 2-hour evidence-based POC curriculum for pediatric residents at 3 different pediatric residencies. At one site, residents exposed to the curriculum performed significantly better on an OSCE for POC than residents who were not yet exposed to the curriculum. In a pre/post survey-based evaluation at all 3 sites, residents exhibited significantly better knowledge and rated their POC abilities significantly better after training, with improvements sustained at 6 months. In a quasiexperimental comparison across time with an untrained control group recruited from 2 of the sites, trained residents exhibited significantly better knowledge and rated their POC abilities significantly better than untrained residents at 6 months. These findings provide a preliminary signal of the potential effectiveness of this intervention. In addition, participating residents across all 3 sites rated the curriculum highly in importance, value, and usefulness, indicating acceptability. We also found that the curriculum could be implemented at 3 different sites, in 3 states with different sociopolitical climates, and was easily tailored to different schedules and educational formats, all of which attest to the curriculum’s feasibility as a generalizable intervention.

This curriculum is innovative because prior curricula on options counseling in the literature are not tailored toward the developmental needs of adolescents. [7–10] Our curriculum was informed by qualitative interviews with 50 individuals who experienced pregnancy before the age of 20 years old, and recommendations from multidisciplinary community clinicians who serve pregnant adolescents. [12,13] Advocates for Youth offers an online curriculum in adolescent reproductive health, including POC, that was initially developed by Physicians for Reproductive Health; however, this curriculum is not specifically for pediatric residents, who might require more experiential training—such as the role-plays and standardized patient simulations included in our curriculum—to maximize gains in skills, given low clinical exposure to POC during residency. [14].

Pediatricians are crucial front-line individuals in supporting adolescents’ health and autonomy and must be trained to deliver POC. The AAP recommends that pediatricians should offer pregnant adolescents comprehensive POC, or if unable to do so (for instance, due to conscientious objection), promptly transfer care to someone who can deliver such counseling. [5] The American Board of Pediatrics includes pregnancy on the pediatrics board certification exam as content that all board-certified pediatricians should understand. [15] Implementation of our curriculum in pediatric residency programs supports and actualizes these critical recommendations.

Though little is known about the counseling practices of pediatricians, known deficits in POC in adult family planning contexts indicate that current clinical training is insufficient. In a survey of adult patients at family planning clinics across the southern United States, only 10% of patients with a positive pregnancy test reported that their clinician performed comprehensive POC that addressed all pregnancy options. [16] Notably, patients whose clinicians had received formal training in POC were more likely to discuss all pregnancy options, which in turn increased the likelihood of patients rating the counseling as high-quality. [16] A separate analysis from the same study also revealed critical health equity concerns in reproductive health outcomes. Patients identifying as Black who wanted referrals for adoption or abortion were less likely to receive such referrals than patients identifying as non-Black. [17] Similarly, one study of clinicians at federally funded family planning clinics in the United States found a significantly smaller proportion of clinicians offered referrals for abortion compared to adoption. [18] These deficits in POC performance attest to the need for interventions such as our curriculum to better train the US medical workforce in comprehensive and unbiased POC. In addition, research on how quality of POC affects adolescent reproductive health outcomes is needed.

Implementing our curriculum is timely given the recent destabilization of abortion care in the United States following the removal of federal abortion protections. [19] Adolescents were already likely to present later in pregnancy for abortion care compared to older individuals. [11] They now face additional challenges to accessing abortion when desired (i.e. logistical challenges of traveling across states if needed, exclusion from self-managed or telehealth abortion protocols), and thus may be disproportionately impacted by abortion restrictions. [20–22] Training clinicians to support adolescents’ reproductive autonomy through patient-centered counseling, timely connection to subsequent care, and support navigating local resources is more important than ever before.

Future directions for this curriculum include a cluster-randomized controlled trial across more pediatric residencies. Such a trial would address limitations in this study, such as absence of randomization procedures that would allow causal inference about training program effects. Such a trial could enroll a greater number of participants to increase power and assess effectiveness over a greater number of sites. Furthermore, this study relied on subjective self-report on surveys to assess changes in skills, although the single-site OSCE does attest to the potential of the curriculum to impact objective demonstration of skills. Notably, the OSCE was only performed in the immediate posttraining period, while survey results demonstrated durability of changes up to 6 months after training. The planned future trial would ideally evaluate for objective differences in skills (for instance, by OSCE) both in the immediate postsetting and at a minimum of 6 months after training, and also assess for changes in clinician behavior and patient outcomes (e.g., perceived quality of counseling, congruence of patient desires and provision of referrals to care). We did not concurrently administer the OSCE and survey instruments in the same individuals, so cannot evaluate whether findings on these metrics are correlated; however, this is a future direction. The present study findings are also limited by substantial attrition in survey responses from the presurvey to subsequent time points, though the OSCE findings are not affected by such attrition and nevertheless attest to a positive immediate effect of the curriculum.

### Conclusion

A 2-hour evidence-based adolescent-centered POC curriculum for pediatric residents was acceptable to residents and feasible to implement at 3 residencies. The curriculum resulted in pre/post improvement in POC performance in an OSCE at one site, and pre/post improvements in knowledge and self-rated skills durable at 6 months across 3 sites. Further research is needed to demonstrate the effectiveness of this curriculum on behavioral and clinical outcomes. Pediatric residencies should consider timely implementation of curricula in adolescent-centered POC for their residents to align with national recommendations and reduce barriers to prenatal or abortion care, especially given the evolving sociopolitical climate related to abortion.

## Supplementary Material

Supplementary Table 6

Appendix 1

Appendix 2

Appendix 3

## Figures and Tables

**Table 1:** Sample demographics for single-site (site #1) observed structured clinical exam (n = 25)

	Pretraining group	Posttraining group

n	13	1
Age (median, (IQR))	27 (27–31)	27 (27–29)
Gender		
Man	3 (23%)	6 (50%)
Woman	10 (77%)	6 (50%)
Race & ethnicity		
White non-Hispanic	11 (85%)	8 (67%)
White Hispanic	2 (15%)	2 (17%)
Asian non-Hispanic	0 (0%)	2 (17%)
Prior POC training		
No	11 (85%)	12 (100%)
Yes	2 (15%)	0 (0%)

Residents at Site #1 were randomly assigned to 2 independent groups to complete an OSCE pre-POC training or post-POC training.

IQR = interquartile range.

**Table 2:** Scores for single-site observed structured clinical exam

	Pretraining group Median (IQR)	Posttraining group Median (IQR)

Overall performance score	9 (8, 10)	13 (13, 14)*
Subscore for disclosing pregnancy test	1 (1, 2)	3 (3, 3)*
Subscore for discussing options	2 (1, 3)	4 (4, 4)*
Subscore for ensuring support & safety	0 (0, 1)	1 (1, 2)*
Subscore for planning next steps	2 (1, 2)	2.5 (2, 3)*
Subscore for communication skills	3 (2, 3)	3 (3, 3)

IQR = interquartile range.

* *p* < .05.

**Table 3:** Sample demographics for multisite survey evaluation

	Training group			Control group	
	Presurvey	Immediate postsurvey	6-Month survey	Presurvey	6-Month survey

n	79	65	40	140	94
Median age (IQR)	28 (27, 29)	28 (27, 30)	28 (27, 29)	29 (28, 31)	29 (28, 31)
Gender					
Man	23 (29%)	18 (28%)	11 (28%)	24 (17%)	21 (22%)
Woman	56 (71%)	44 (68%)	27 (69%)	114 (81%)	69 (73%)
Unspecified	0 (0%)	3 (5%)	2 (5%)	2 (1%)	4 (4%)
Prior POC training					
Yes	13 (16%)	10 (15%)	9 (23%)	23 (16%)	17 (18%)
No	66 (84%)	55 (85%)	29 (73%)	114 (81%)	73 (78%)
Unspecified	0 (0%)	0 (0%)	2 (5%)	3 (2%)	4 (4%)
Baseline postgraduate year					
1	52 (66%)	41 (63%)	28 (70%)	25 (17%)	17 (18%)
2	14 (18%)	11 (17%)	7 (18%)	26 (16%)	18 (19%)
3	6 (8%)	6 (9%)	2 (5%)	34 (24%)	17 (18%)
4	5 (6%)	5 (8%)	2 (5%)	22 (16%)	17 (18%)
5	2 (3%)	2 (3%)	1 (3%)	16 (11%)	14 (15%)
6	0 (0%)	0 (0%)	0 (0%)	11 (8%)	9 (10%)
7	0 (0%)	0 (0%)	0 (0%)	4 (3%)	2 (2%)
Unspecified	0 (0%)	0 (0%)	0 (0%)	2 (1%)	0 (0%)
Race & ethnicity					
White non-Hispanic	54 (68%)	46 (71%)	29 (73%)	87 (62%)	57 (61%)
White Hispanic	3 (4%)	3 (5%)	1 (3%)	8 (6%)	6 (6%)
Black non-Hispanic	3 (4%)	1 (2%)	0 (0%)	11 (8%)	7 (7%)
Asian non-Hispanic	11 (14%)	8 (12%)	7 (18%)	19 (14%)	14 (15%)
Other	8 (10%)	7 (11%)	3 (8%)	13 (9%)	6 (6%)
Unspecified	0 (0%)	0 (0%)	0 (0%)	2 (1%)	4 (4%)
Site state					
Pennsylvania (Site #1)	31 (39%)	24 (37%)	20 (50%)	0 (0%)	0 (0%)
North Carolina (Site #2)	21 (27%)	17 (26%)	8 (20%)	19 (14%)	4 (4%)
Indiana (Site #3)	27 (34%)	24 (37%)	12 (30%)	121 (86%)	90 (96%)

IQR = interquartile range.

**Table 4:** Results of premonth, postmonth, and 6-month surveys for multisite evaluation

	Training group			Control group	
	Presurvey Median (IQR)	Immediate postsurvey Median (IQR)	6-Month survey Median (IQR)	Presurvey Median (IQR)	6-Month Survey Median (IQR)

Knowledge score^a^	57% (24% 83%)	90% (81%, 95%)	86% (57%, 90%)	57% (47%, 81%)	67% (48%, 86%)
Self-rated knowledge of pregnancy resources^b^	2 (1, 2)	3 (3, 4)	3 (2, 3)	2 (1, 2)	2 (2, 3)
Overall discussion score^c^	8 (7, 9)	12 (10, 12)	10 (9, 12)	8 (6, 9)	9 (8, 10)
Self-rating to discuss parenting^b^	3 (2.5, 3)	4 (4, 4)	4 (3, 4)	3 (3, 4)	3 (3, 4)
Self-Rating to Discuss Abortion^b^	3 (2, 3)	4 (3, 4)	3 (3, 4)	3 (2, 3)	3 (2, 3)
Self-rating to discuss adoption^b^	3 (2, 3)	4 (3, 4)	3 (3, 4)	3 (2, 3)	3 (2, 3)
Overall referral score^c^	6 (5, 8)	10 (9, 12)	9 (8, 11)	6 (5, 8)	7 (6, 9)
Self-rating to refer for prenatal care^b^	2 (2, 3)	3 (3, 4)	3 (3, 4)	2 (2, 3)	3 (2, 3)
Self-rating to refer for abortion^b^	2 (1, 3)	4 (3, 4)	3 (3, 4)	2 (1, 3)	2 (2, 3)
Self-rating to refer for adoption^b^	2 (1, 2)	3 (3, 4)	3 (2, 3)	2 (1, 3)	2 (2, 3)

IQR = interquartile range.

a Based on average of 3 multiple-choice questions assessing knowledge of pregnancy options, state parental consent laws for abortion, and local pregnancy-related resources.

b Based on 3 summed 4-point Likert scale scores (3-worst rating, 12-best rating).

c Based on a 4-point Likert scale (1-worst rating, 4-best rating).

**Table 5:** Adjusted odds ratios (aORs) or beta coefficients (ß) for improvement in scores on postsurvey and 6-month survey compared to presurvey scores in training group

	Immediate postsurvey aOR^a^ or ß (95% CI)	6-month follow-up survey aOR^a^ or ß (95% CI)	Interaction term aOR or ß (95% CI)

Knowledge score	0.29 (0.23, 0.35)*	0.25 (0.17, 0.32)*	0.15 (0.11, 0.19)*
Self-rated knowledge of pregnancy resources	259.41 (55.60, 1210.25)*	20.69 (6.58, 65.06)*	4.24 (2.71, 6.62)*
Overall discussion score	52.91 (19.52, 143.44)*	16.69 (6.34, 43.93)*	4.01 (2.60, 6.16)*
Self-rating to discuss parenting	38.56 (11.66, 127.48)*	18.89 (5.74, 61.98)*	5.93 (3.44, 10.22)
Self-rating to discuss abortion	70.25 (20.24, 243.80)*	11.51 (3.89, 34.04)*	4.01 (2.50, 6.45)
Self-rating to discuss adoption	22.98 (8,85, 59.68)*	8.00 (3.17, 20.17)*	3.12 (2.02, 4.84)
Overall referral score	79.31 (27.96, 224.96)*	19.42 (7.42, 50.86)*	4.72 (3.06, 7.27)*
Self-rating to refer for prenatal care	30.57 (11.71, 79.77)*	13.68 (5.30, 35.28)*	3.90 (2.53, 6.00)
Self-rating to refer for abortion	88.47 (26.71, 292.99)*	21.96 (7.29, 66.14)*	5.81 (3.54, 9.52)
Self-rating to refer for adoption	53.25 (19.05, 148.83)*	11.78 (4.71, 29.45)*	4.20 (2.70, 6.52)

CI = confidence interval.

* *p* < .05 for comparisons by mixed-effect regression or ordinal logistic regression with presurvey data.

a Adjusted for site, baseline reported experience of prior training of pregnancy options counseling, and baseline PGY year.
