# Supplementary material for: Evaluation of a Pregnancy Options Counseling Curriculum for Pediatric Residents
Source: J Adolesc Health. Author manuscript; Available in PMC 2025 Mar 3. (PMC11875686; doi:10.1016/j.jadohealth.2024.11.003)
Supplement: Supplementary Table 6 [file NIHMS2055353-supplement-Supplementary_Table_6.docx]

**Supplementary Table 6. Beta Coefficient or Adjusted Odds Ratios (aORs) for Intervention Effect Demonstrating Superior Performance in Training Group Compared to Control Group**

|  | **Intervention Effect**^a^  **aOR or ß (95% CI)** |
| --- | --- |
| Knowledge Score | 0.15 (0.11, 0.19)* |
| Self-Rated Knowledge of Pregnancy Resources | 4.24 (2.71, 6.62)* |
| Overall Discussion Score  Self-Rating to Discuss Parenting  Self-Rating to Discuss Abortion  Self-Rating to Discuss Adoption | 4.01 (2.60, 6.16)*  5.93 (3.44, 10.22)*  4.01 (2.50, 6.45)*  3.12 (2.02, 4.84)* |
| Overall Referral Score  Self-Rating to Refer for Prenatal Care  Self-Rating to Refer for Abortion  Self-Rating to Refer for Adoption | 4.72 (3.06, 7.27)*  3.90 (2.53, 6.00)*  5.81 (3.54, 9.52)*  4.20 (2.70, 6.52)* |

^a^ Adjusted for site, baseline reported experience of prior training of pregnancy options counseling, and baseline PGY year

*p<0.05 for interaction term between time point and training group in mixed-effect regression or ordinal logistic regression
