# Supplementary material for: Evaluation of a Pregnancy Options Counseling Curriculum for Pediatric Residents
Source: J Adolesc Health. Author manuscript; Available in PMC 2025 Mar 3. (PMC11875686; doi:10.1016/j.jadohealth.2024.11.003)
Supplement: Appendix 1 [file NIHMS2055353-supplement-Appendix_1.docx]

**Appendix 1 Pregnancy Options Counseling Curriculum: Facilitation Guide**

**Timing**

**Large group**

00:00 – 00:05 Arrivals

00:05 - 00:10: Introduction, review of learning objectives and format of training

00:10 - 00:35: Discussion/Didactics

**Break into small groups**

00:35 - 00:47: Standardized patient encounter #1

00:47 – 00:59 Standardized patient encounter #2

00:59 - 01:09 Offer ten minute break

01:09 - 01:21 Standardized patient encounter #3

01:21 - 01:33 Standardized patient encounter #4

01:33 - 01:45 Standardized patient encounter #5

**Resume large group**

01:45 - 02:00 Reflections and wrap up

~~~~~~~~~~~~~~~~~~~~~~~~~~~~~~~~~~~~~~~~~~~~~~~~~~~~~~~~~~~~~~~~~~~~~~~~

00:00 – 00:05 Arrivals

00:05 - 00:10: Introduction, review of learning objectives and format of training

**Introduction, review of learning objectives and format of training**

Learning objectives

By the end of the training, residents should be able to:

1. Use sensitive and appropriate communication skills and language to disclose a positive pregnancy test and discuss pregnancy options with a pregnant adolescent patient
2. Identify and name appropriate pregnancy-related resources and referrals
3. Apply knowledge of pregnancy options, including pertinent laws, in clinical scenarios

Expectations

-It is the ethical standard of care for doctors to be able to provide, in the setting of a positive pregnancy test:

1. Counseling around all 3 options: adoption, abortion, and parenting OR
2. Referral to providers who can provide counseling about all 3 options

-Endorsed by the American Academy of Pediatrics

-If for any reason you feel like you do not have sufficient knowledge to perform counseling on all three options or are too busy or have a moral objection, then it is your responsibility as a provider to arrange for a different provider to perform appropriate counseling and referrals.

--Note: the healthcare provider offering options counseling should counsel about all options in one session to avoid bias; it is not appropriate for a healthcare provider to counsel on just one or two options, then hand off for counseling about remaining options.

Format of training

-First we are going to discuss pregnancy options counseling.

-Then we are going to split off into two groups in which each resident will have time to practice with a standardized patient.

-Finally we will reconvene and talk about what we have learned.

-The goal is for you to get comfortable “getting the words out” and for you to know where you can find resources in the future if you are faced with disclosing a positive pregnancy test.

00:10 - 00:35: Discussion/Didactics

**Values clarification exercise (~7 minutes)**

We will take a moment to reflect on how our personal values and biases might impact our options counseling and ability to engage with adolescents’ pregnancy decisions. Please everyone take a moment to read Handout 1 [see end of document].

Discussion questions (no need to utilize all of these):

What thoughts or feelings came up for you when reviewing these scenarios?

How do our personal values impact our potential to perform unbiased options counseling?

How do you navigate internal conflicts when interacting with patients?

What strategies can we use to practice emotional intelligence in patient encounters?

**Discussion/Didactics**

**Disclosure of pregnancy test:** Please inform patients privately that a pregnancy test will be ordered. If one has already been ordered (i.e. by nurse protocol), please ask the patient privately if they are aware that a pregnancy test has been ordered.

**Suggested language for disclosure of pregnancy:** “Your pregnancy test is positive, which means that you are pregnant.”

**Suggested language for parenting:** “Become a parent” (as opposed to “keep the baby”)

**Suggested language for adoption:** “Make a plan for an adoption” (as opposed to “give the baby up for adoption”)

**Suggested language for abortion:** “have an abortion” (as opposed to “terminate the pregnancy” or “end the pregnancy”). It can be appropriate to use a euphemism for abortion on a case-by-case basis if the patient uses one in order to reflect back their language and ensure their comfort, but the concept of abortion should be introduced as “abortion” at least initially to ensure clarity. More neutral euphemistic language such as “termination” is likely appropriate to reflect, but we would advise avoiding reflection of highly values-laden language such as “murder” or “kill.”

**Information about decision-making:**

-Balance allowing time to make a good decision (i.e. a decision that the patient feels comfortable with) with timely decision-making

-Adolescents are more likely to present for both prenatal care and abortion care at later gestations

-Decision-making assistance referrals (online options counseling workbook, All Options Talkline)

-Goal is not for us to be experts in pregnancy options but to be able to present basic information to patients, emotionally support them, and refer them for more information

**Information about prenatal care and becoming a parent:**

-Discuss appropriate referrals for prenatal care

-Discuss appropriate referrals for governmental and charitable assistance for pregnant people and parents

-Discuss appropriate prenatal counseling such as provision of vitamins, cautioning about tobacco/drugs/alcohol, reviewing medications, importance of timely prenatal care

-Provide handouts with resources and referrals for healthcare provider reference [see attached]

-Discuss availability of discharge education about pregnancy in electronic medical record

**Information about adoption:**

-Discuss open versus closed adoption plans

-People choosing adoption can choose the level of “openness” of the adoption, but the agreement is not legally enforceable and is not “co-parenting.”

-Adoption is a legally binding agreement, officially made no earlier than 48 hours after birth

-People choosing adoption can choose the adoptive parents in many circumstances

-Adoption is not foster care or child welfare

-Discuss appropriate referrals for adoption locally

-As with all options, neutrality in discussing adoption is important (i.e. can be problematic to say “What an amazing brave thing” or “You don’t have to do this”)

**Information about abortion:**

-Discuss appropriate referrals for abortion care

-Discuss providing only reliable referrals given the ubiquity of crisis pregnancy centers

-Medical abortion (pill) available in PA to 11 weeks EGA

-Surgical abortion available in PA to 23.6 weeks EGA

-Discuss importance of timely abortion services (i.e. although procedure is safe at all gestations, it is less complicated, briefer, and less expensive in the first trimester).

-Discuss PA parental consent law for abortion and judicial bypass process

-Discuss insurance coverage limitations and sources of financial assistance

**Managing confidentiality and parental relationships:**

-Confidential disclosure and discussion of results

-Adolescents do not need parental consent for pregnancy options counseling, prenatal care, or placement for adoption

-Caveats to confidentiality: mandatory reporting scenarios, risk of harm to self or others

-Important to screen for partner safety and family/household safety

-OK to encourage involvement of parents when appropriate

-Should ask if the patient would like someone else (a support person such as a parent) to be present for further discussion of the results, and if the patient would like you to help them disclose the results to the other person

-Often but not always helpful for next steps and follow up if adolescent able to safely disclose pregnancy to parents, but adolescent can decline to disclose even if no safety concerns

-If adolescent not able to discuss with parents due to safety concerns, consider involving social work and/or domestic violence resources where appropriate for safety planning for home environment

-Consider maintaining confidentiality in documentation (i.e. confidential notes after encounter)

**Universal referrals for mental health resources:**

-Ideally provide patients with information about local crisis services (Resolve in Pittsburgh area)

-“For some people, but not everyone, becoming pregnant can be a major life change, and life changes situations can be stressful. For that reason, we give everyone we see who is pregnant this card in case they or someone they know experience a crisis or emergency related to their mental health.”

-Can also discuss this referral in the context of partner violence or other safety concerns: “Some people I care for do not have a network of supportive people around them or have concerns about their safety. There is a great resource that I like to tell everyone about.”

**Importance of plan for next steps:**

Adolescents may benefit from assistance developing a concrete plan of action for their next steps. This could include providing appropriate referrals and reviewing the referrals with them. This could also include making plans to disclose the pregnancy to important people in the adolescent’s life, such as parents where safe. For adolescents who are undecided about the pregnancy, it is particularly important to establish that they have someone to check in with about their intentions, such as a trusted primary care provider.

Concrete steps that providers can take include: identifying providers closest to a young person’s home or bus line (using Google Maps or another service), staying in the room with a young person while they call for an appointment, or being present during a conversation with parents/guardians.

**Case discussion**

**Vignette to share with audience:** Kayla is a 16-year-old girl with no significant medical history.  Kayla has had intermittent abdominal pain for the last few days, and today she had 2 episodes of vomiting, so she came to the ED for evaluation.  Her LMP was 8 weeks ago. A pregnancy test is performed as part of her ED work-up and is positive.

**Discussion prompts** (no need to utilize all of these):

-How would you tell her about the pregnancy test result?

-What else would you like to know about Kayla?

-What else would you like to discuss during this conversation?

-What if she’s very undecided or ambivalent?

-What are some strategies for communicating effectively with Kayla during this conversation?

**Background information (revealed through audience questions as applicable):** *Kayla enjoys playing tennis and reports this as one of her biggest strengths. She lives at home with her mother, father, and older brother. She feels safe at home and has no concerns for violence. In addition to tennis, Kayla is active in her school’s theater department and spends time volunteering at the local library. She is hoping to go to college after high school.  She sometimes feels “stressed” about her schoolwork, but denies any frank symptoms of anxiety or depression. She has no history of alcohol, tobacco, or illicit drug use. She has been sexually active with her boyfriend of 8 months, and she reports that they use condoms most of the time.*

**Objectives to cover:**

-Ensure confidentiality

-Check in emotionally (“How are you feeling about this?”)

-Assess expectations (“Did you think that you might be pregnant?”)

-Assess experiences (“Have you ever been pregnant before?”)

-Introduce options (“Are you aware of what your options are regarding the pregnancy?”)

-Explain options (“There are three options, which include…”)

-Addressing ambivalence (pros and cons, planning for next 6-12 months)

-What to do patient asks “What do you think I should do?” Support them in making their own decision

-Assess support network

-Assess home/family safety

-Assess partner safety

-Communication strategies

-allow silence

-OARS model (open ended questions, affirmations, reflective listening, summary

reflections)

1. -“Ask Tell Ask” model (Information from the American Medical Association about Ask Tell Ask: [10.1001stepsforward.2017.0048supp3.docx (live.com)](https://view.officeapps.live.com/op/view.aspx?src=https%3A%2F%2Fedhub.ama-assn.org%2Fdata%2Fjournals%2Fsteps-forward%2F937327%2F10.1001stepsforward.2017.0048supp3.docx&wdOrigin=BROWSELINK))
2. -NURSE model (name, understand, respect, support, explore) (Information about NURSE model from Vital Talk: [Responding to Emotion: Respecting - VitalTalk](https://www.vitaltalk.org/guides/responding-to-emotion-respecting/))

-Avoiding bias

-Pregnancy-related immediate medical care (prenatal vitamins, review of medications, avoidance of illicit drugs, tobacco, and alcohol, testing for sexually transmitted infections)

-Provide appropriate referrals and identify next steps

**Break into small groups**

00:35 - 00:47: Standardized patient encounter #1

00:47 - 00:59 Standardized patient encounter #2

00:59 - 01:09 Offer ten minute break

01:09 - 01:21 Standardized patient encounter #3

01:21 - 01:33 Standardized patient encounter #4

01:33 - 01:45 Standardized patient encounter #5

**Personnel**

Small groups should consist of up to five participants, plus a facilitator and one or more actors to portray standardized patients. The standardized actors should not be the participants, but should be separate, trained individuals. We conduct a one-to-two hour training and practice session of all standardized actors and facilitators prior to the event to ensure adequate preparation. The small group facilitator should be a trained individual with expertise in adolescent medicine and/or pregnancy options counseling.

**Facilitating Discussion of Standardized Patient Encounters: General Discussion**

There is no need to cover all these questions. These are prompts in case of slow participation. The priority in discussion is to debrief and feedback about each encounter, rather than group discussion of the prompts.

**For the participant:**

-   *What are your initial reactions?  What do you think went well? Anything you would want to do differently?*

- *Are there any questions that you would like to ask the standardized patient about this experience?*

**For the audience:**

*-    What did you notice about the way they disclosed the pregnancy test results?*

*-    What did you notice about the general structure/format of the discussion?*

*-    How do you think they created safe space and reduced stigma in pregnancy decisions?*

*-    Did you feel they kept discussions free from bias?  How did they accomplish this?*

*- What did you notice about how they established a plan for next steps?*

**Cases**

**Standardized Patient Case #1: Not surprised, ambivalent**

**Learning objectives:**

- Addressing ambivalence
- Working with patients with strong anti-abortion attitudes
- Describing and referring for adoption
- Discussing open versus closed adoption

**Information for Participant:** Julia is a 16 year old girl with no significant medical history.  Julia has had intermittent abdominal pain for the last few days, and today she had 2 episodes of vomiting, so she came to the ED for evaluation. Her LMP was 8 weeks ago. A pregnancy test is performed as part of her ED work-up and is positive. She came to the ED by herself.

**Situational background for Standardized Patient**: Julia is not surprised by the positive pregnancy test. She had already taken 3 home pregnancy tests that were all positive, so she already thought she was pregnant, but she wanted to go to a doctor to confirm it.   She is ambivalent about her options. She doesn’t want to have a baby but is against abortion. She chooses adoption.

***Character Background for* Standardized Patient***: Julia enjoys playing with her dog and singing.  She says that singing is one of her biggest strengths. She lives at home with her father and younger sister Erin.  She feels safe at home and has no concerns for violence. She would like to become a professional singer or music teacher.  She is stressed out about trying to take care of her boyfriend, who is not compliant with his Type 1 diabetes therapies, but she is not anxious or depressed.  She has no history of alcohol, tobacco, or illicit drug use. She has been sexually active with her boyfriend of 6 months, and she reports that sometimes they forget condoms.  She identifies herself and her family as Christian. She has been raised in a politically conservative household. She personally believes that abortion is murder, and thinks it is very wrong. She is open to the possibility of adoption because she thinks it would be a beautiful gift to a family that wants children, and she also she has a friend from school who is adopted. She would prefer to have an open adoption, rather than a closed adoption.*

*Sample dialogue for* **Standardized Patient***: “My family believes that abortion is murder.  I can’t do that. I don’t believe in that.” “I don’t know if I could go through all that to give up the baby, but I don’t think I can raise a baby right now, either.” “If I give away the baby, will I never get to meet him or her?”*

Discussion prompts:

- How would you react if a patient told you that they believe abortion is murder?
- Is it necessary to address abortion as part of options counseling if a patient indicates that they don’t believe in it?
- How much information is necessary to provide about adoption during options counseling?

**Standardized Patient Case #2: Surprised, distressed**

**Learning objectives:**

- Communicating with a very anxious patient
- Addressing relationship and family safety
- Describing the medical risks and safety of abortion
- Discussing parental consent laws and judicial bypass regarding abortion

***Note for facilitator****: If participant does not ask parent to leave before starting to talk to the patient about their pregnancy diagnosis, please halt and “rewind” the roleplay.*

**Information for Participant**: Mackenzie is a 16-year-old girl with no significant medical history.  Mackenzie has had intermittent abdominal pain for the last few days, and today she had 2 episodes of vomiting, so she came to the ED.  Her LMP was 8 weeks ago. A pregnancy test is performed as part of her ED work-up and is positive. Her mother is in the room with her.

**Situational background for** **Standardized Patient (Mackenzie)**: Mackenzie is surprised.  She was not planning a pregnancy and is very upset by the result.  She is terrified of her parents’ and partner’s reactions, as she anticipates that they will be angry and disappointed in her.  She feels like pregnancy and parenting would derail her life and interfere with her goals. She is worried about abortion but is reassured by the provider explaining that abortion is safe.  She chooses abortion.

**Situational background for** **Standardized Patient (Mackenzie’s mother):** Mackenzie’s mother is concerned about her daughter’s symptoms and attentive to her daughter while they are in the room together. She will immediately agree to leave the room when asked.

***Character Background for*** **Standardized Patient (Mackenzie)***: Mackenzie is a straight A student and on the Honor Roll, and says that her biggest strength is science. She wants to go to college to study psychology on a scholarship. She also enjoys swimming, but is not on the swim team. She lives at home with her mother and father, and two younger sisters.  She feels safe at home. Her boyfriend is an athlete and expects to get a sports scholarship, and they have talked about going to college together. She has tried beer (1-2 drinks) at parties, but no tobacco or illicit drug use. She puts a lot of pressure on herself to be a role model for her siblings.  She has been sexually active with her boyfriend of 1 year, and they always use condoms. Although she is afraid that her parents and partner will be upset, she does not think they will be violent towards her but she worries that her boyfriend might break up with her.*

**Sample dialogue for** **Standardized Patient (Mackenzie)**: “My parents are going to kill me.”  “I’m scared of what my boyfriend will do.” “I’ve read a lot of things about abortion -- that it can cause breast cancer, that it can make it so you can’t have kids later, that it can cause weird infections, even that it can kill you.  I don’t think I could do that.” “If I have an abortion, do my parents have to find out?”

Discussion prompts:

- How would you react if a patient told you that her parents are going to kill her?
- What would you do if a patient asked you a question about abortion and you don’t know the answer?
- How would you react if a patient told you they are interested in having an abortion?
- What are some strategies for communicating with a patient who is anxious and upset?

**Standardized Patient Case #3: Surprised, interested in parenting**

**Learning objectives:**

- Communicating with a teen interested in parenting
- Prenatal counseling
- Referring for prenatal care

***Note for facilitator****: If participant does not ask parent to leave before starting to talk to the patient about their pregnancy diagnosis, please halt and “rewind” the roleplay.*

**Information for Participant**: Lena is a 16-year-old girl with no significant medical history.  Lena has had intermittent abdominal pain for the last few days, and today she had 2 episodes of vomiting, so she came to the ED for evaluation.  Her LMP was 8 weeks ago. A pregnancy test is performed as part of her ED work-up and is positive. Her mother is in the room with her.

**Situational background for Standardized Patient (Lena):**  Lena is surprised. She had no idea she could be pregnant and is shocked at first.  She is initially ambivalent but with more consideration she thinks that having a baby could be a good experience for her because she has strong social supports and had always planned on having a large family, and her mother also had her when she was 17. She also likes the idea of having a baby that will unconditionally love her. She chooses parenting.

**Situational background for** **Standardized Patient (Lena’s mother):** Lena’s mother is highly affectionate toward her daughter while they are in the room together. They appear to have a close relationship. When asked to leave the room initially, Lena’s mother is at first resistant (briefly), before being agreeable to leave after being asked a second or third time.

***Character Background for Standardized Patient (Lena):*** *Lena thinks that her biggest strength is she is a very caring person.  She is not sure that she will go to college, but wants to either work at a daycare or be a nurse. She lives at home with her mother, who had her when she was 17.  She feels safe at home and has no concerns for violence. She had sex two times a couple months ago with her friend’s older brother, who is 19. She drinks alcohol about twice a month (two drinks maximum) and has smoked marijuana three times. No tobacco use.  She thinks her mother, grandparents, and best friends would be supportive of her pregnancy. She is not sure how the father will react, but doesn’t think he would be violent or unsafe. She wants to have a large family and thinks that being a mother would give her a sense of purpose.*

***Sample dialogue for Standardized Patient (Lena):*** *“Are you sure? Are you really sure? [regarding the pregnancy test results]” “My mom had me when she was 17.  She was a wonderful mother. If she can do it, I know I can do it too.”*

***Sample dialogue for Standardized Patient (Lena’s mother):*** *“Anything you can say in front of my daughter you can say in front of me.”*

Discussion prompts:

- How would you react if a patient told you they are interested in becoming a parent?
- What information do you think it would be important to share with a patient who is interested in becoming a parent?
- How would you address the presence of a parent in the room? What if the parent did not want to leave or allow confidential discussion?

**Standardized Patient Case #4: Not surprised, distressed**

**Learning objectives:**

- Assessing relationship safety
- Addressing potential reproductive coercion
- Addressing privacy of abortion regarding partner

***Note for facilitator****: If participant does not ask partner to leave before starting to talk to the patient about their pregnancy diagnosis, please halt and “rewind” the roleplay.*

**Information for Participant:** Bridget is a 16-year-old girl with no significant medical history.  Bridget has had intermittent abdominal pain for the last few days, and today she had 2 episodes of vomiting, so she came to the ED.  Her LMP was 8 weeks ago. A pregnancy test is performed as part of her ED work-up and is positive. Her boyfriend Steve is with her.

**Situational background for Standardized Patient (Bridget)**:  Bridget is not surprised. She had missed her last period so she had taken a home pregnancy test which was positive, but she was hoping it was a mistake.  She came to the hospital because she was scared about being pregnant. She suspects that her partner might have tampered with the condoms because he is always talking about how great it would be to have a baby, but Bridget does not want to be a parent.  She does not want her partner to know about the positive pregnancy test. She chooses abortion. She thinks her parents will be supportive of her having an abortion.

***Character Background for*** **Standardized Patient (Bridget)***: Bridget thinks that her biggest strength is that she is good at drawing.  She wants to go to college for criminal justice. She lives at home with her mother, father, and older brother. She feels safe at home.  She has had a boyfriend for 6 months. They have been sexually active for 2 months and always use condoms. Her boyfriend is 18 years old and talks about wanting to be a father a lot.  He is not violent and does not pressure her for sex, but he can be suspicious and controlling, like going through her phone to see if she is texting other guys. She is angry at her boyfriend and is worried that he might have poked holes in the condom. She has concerns about her boyfriend and does not view him as a viable long-term partner, but does not consider herself to be in an “abusive relationship” per se. She tried alcohol one time, and smokes marijuana a couple times a week.  No tobacco use. She thinks her parents would be supportive of her having a baby but she does not want to have a baby with this boyfriend and does not think now is the right time because she wants to focus on graduating from high school.*

***Character Background for Standardized Patient (Steve):*** Steve appears protective, even defensive, of Bridget while they are in the room together. He may place one hand on her shoulder. When asked to leave, he is initially resistant and unsure. When asked a second time, he asks Bridget if she is okay with him departing the room, and she affirms that it is. He grudgingly exits the room.

**Sample dialogue for** **Standardized Patient**: “I think he did something to the condoms.” “Do I have to tell him if I’m having an abortion?”

Discussion prompts:

- How would you react if a patient described a relationship like this to you?
- What would you do if a patient was concerned about a partner sabotaging birth control?
- What is important to discuss with a patient who is concerned about confidentiality?

**Standardized Patient Case #5: Surprised, ambivalent**

**Learning objectives:**

- Assessing ambivalence
- Balancing allowing time to decide with importance of timely decision-making
- Referring to further options counseling resources
- Importance of reinforcing that the decision is the pregnant person’s and not to be made by parents or healthcare providers

**Information for Provider**: Karina is a 16-year-old girl with no significant medical history.  Karina has had intermittent abdominal pain for the last few days, and today she had 2 episodes of vomiting, so she came to the ED for evaluation.  Her LMP was 8 weeks ago. A pregnancy test is performed as part of her ED work-up and is positive. She came to the ED by herself.

**Situational background for** **Standardized Patient**:  Karina is surprised. She had thought she was being really careful.  She had never really thought about what she would do if she got pregnant.  She is very ambivalent. She was the product of a teenage pregnancy herself, and being inspired by her own mother’s strength, she thinks she could be a good parent.  She has good social supports. She has mixed feelings about abortion; her family is religious and she is concerned that they would look down on that decision, but she herself does not think abortion is wrong.  She is interested in adoption because she thinks it would be more acceptable to her family, and it would still allow her to finish school, but she is worried that it would be too difficult to place for adoption after carrying through with an entire pregnancy.  She chooses to keep thinking about her options.

***Character Background for*** **Standardized Patient***: Karina thinks that her biggest strength is that she is good at playing the piano.  She wants to finish high school, but has not thought much about what she wants to do after that. She thinks that she will either go to community college or get a job working at a restaurant or in retail sales. She sees herself becoming a parent someday. She is not sure if she wants to have a baby now, however, and is not sure how becoming a parent will impact her ability to finish school and participate in extracurricular activities such as diving team and piano.  She lives at home with her mother, sister, brother, and cousin. Her mother was a teen parent and she admires her mother’s strength. She has a good relationship with her boyfriend of 1 year. No safety concerns.  She and her boyfriend use condoms and she thought she was being careful. No alcohol, drug, or tobacco use.*

***Sample dialogue for*** **Standardized Patient***: “I don’t know what to do.”  “Do I need to decide right now?” “If I wanted to have an abortion, do I need to do it right now?” “What do you think I should do?”*

Discussion prompts:

- What would you say if a patient asked you ‘What do you think I should do?’
- What next steps would you arrange with this patient? What resources would you provide?
- What are some strategies to effectively communicate with a very ambivalent patient?

**Standardized Patient Case #6 - Conscientious Objection (Substitute for other cases if needed):**

This encounter is designed to practice a needed handoff of care to another provider who will perform options counseling in the event that the original treating physician cannot perform comprehensive options counseling for any reason, including being too busy or due to conscientious objection.

**Part 1. Disclosing pregnancy test results.**

**Learning objectives:**

-Disclosing positive pregnancy test results to a patient

-Informing a patient of a handoff of care to another provider

**Information for Provider:** Katie is a 16-year-old girl with no significant medical history.  Katie has had intermittent abdominal pain for the last few days, and today she had 2 episodes of vomiting, so she came to the ED for evaluation.  Her LMP was 8 weeks ago. A pregnancy test is performed as part of her ED work-up and is positive. She came to the ED by herself.

**Situational background for Standardized Patient*:*** Katie is not surprised. She had taken a pregnancy test at home and just wanted to be sure today.  She has not thought about her options yet because she feels somewhat overwhelmed. She is concerned that receiving counseling from a second provider will extend the visit significantly when she just wants to go home because she is feeling upset.

***Sample dialogue for SP****: “How long is this gonna take?” “Do I really need to wait for the other doctor?”*

Discussion prompts:

- How would you address informing the patient of a handoff of care to another provider?

**Part 2. Handing off to another provider.**

**Learning objectives:**

- Asking another provider to perform options counseling

**Information for Provider**: You and Sam are the only residents in the ED.  It is a busy day.

**Situational Background for SP**:  Sam is very busy taking care of a patient with an asthma exacerbation and a patient with tracheitis who is headed to the PICU.  When asked to do pregnancy options counseling, Sam is happy to help but is also slightly curious about yet accepting of the other resident’s motivations.

***Sample dialogue for SP****: “What have you talked to this person about and what exactly do you need me to say?”*

Discussion prompts:

- How would you address needing to ask a colleague to perform options counseling?

**Resume large group**

01:45 - 02:00 Reflections and wrap up

Discussion prompts if conversation is slow:

- What do you think will be challenging for you in having these discussions?  Which scenarios do you think would be particularly hard for you?
- How do you think that your own feelings and thoughts might impact your ability to perform options counseling?
- What are some of the biggest takeaways from today’s training? What is your action plan after today’s training?

**Values Clarification Exercise**

Please reflect on how comfortable you are with these individuals’ pregnancy decisions.  This exercise is for you to have a chance to think introspectively about your thoughts and feelings toward pregnancy during adolescence.

You do not need to write down your responses. You do not need to share your responses. Responses will not be collected.

#1 Destiny is a 17-year-old Asian American young woman who is currently parenting her fourteen-month-old son Charles. She lives with her grandmother, who is highly involved in caring for Charles while Destiny attends high school. Destiny is pregnant now and intends to parent her second child. However, her grandmother is upset with Destiny because she feels personally over-extended and does not think their income can support another child.

#2 Olivia is a 14-year-old White young woman who became pregnant by her 14-year-old boyfriend. Olivia thinks she is too young to become a parent, and would like to focus on school for the next several years.  Her parents are politically conservative Christians who believe abortion is murder. Although Olivia herself is not sure what she thinks about abortion, she decides to pursue adoption because she thinks her parents will be happiest with this decision.

#3 Sam is a 16-year-old African American non-binary person who is now 18 weeks pregnant. They first took a pregnancy test, which was positive, at 6 weeks of pregnancy but were hoping the result was a mistake and, despite noticing changes in their body, held out this hope until recently, when they decided to take another test at a local clinic. They would like to pursue having an abortion, and do not want their parents to find out that they were ever pregnant.
