# Supplementary material for: Evaluation of a Pregnancy Options Counseling Curriculum for Pediatric Residents
Source: J Adolesc Health. Author manuscript; Available in PMC 2025 Mar 3. (PMC11875686; doi:10.1016/j.jadohealth.2024.11.003)
Supplement: Appendix 2 [file NIHMS2055353-supplement-Appendix_2.docx]

**Appendix 2 Observed Structured Clinical Exam Case**

**Information for participant:** Kayla Johnson is a 16 year old girl with no significant past medical history. She came to the ED by herself. She had had intermittent nausea, vomiting, and abdominal pain for the past 3 days, and so she decided to present to the ED. Her LMP was 8 weeks ago. She has already disclosed to you that she is sexually active with her boyfriend, who has been her only sexual partner. You ordered a urine pregnancy test as part of her medical workup. It just resulted positive.

Provide the patient with her pregnancy test results and perform pregnancy options counseling. No exam is to be done today.

Objectives of the case

1. Disclose pregnancy test results
2. Perform pregnancy options counseling

**Situational background for standardized patient:** You are not particularly worried about your symptoms, which you think are due to a “stomach bug,” and you came to the ED to get medication to help you feel better. The possibility of being pregnant has not seriously crossed your mind because you usually use condoms with your boyfriend. When you learn about the pregnancy, you feel surprised and overwhelmed. You are ambivalent about your pregnancy options and remain undecided after counseling, although you are grateful for options counseling and resources provided.

**Detailed background:** You see your greatest strength as being a good listener and friend. You are a sophomore in high school and an A/B student. You aren’t sure what you want to do after graduation. You have lived in Pittsburgh your whole life. You live with your father who is a construction worker and who is politically conservative and opposed to abortion. He is supportive and a positive influence in your life. You are not sure of your own political beliefs, but are not categorically opposed to abortion. Your boyfriend is also 16 and attends your high school. You like him because he makes you laugh. You feel safe with him. However, you are concerned that he is not very committed and you don’t see yourself in a long-term relationship with him. You have been sexually active with him for 3 months and usually use condoms.

**Essential dialogue:**

-Pause for a long time (at least 10 seconds) upon learning the pregnancy diagnosis

-Question the diagnosis: **“Are you sure? What if there was some kind of mistake?”** Accept initial affirmation that the test is accurate.

-When presented with options, ask, **“What do you think I should do?”**

-At some point in options counseling, ask “**Do I have to decide right now?”**

-State **“My father is going to kill me.”** When asked about safety, clarify that you have no safety concerns, but are worried about upsetting or disappointing your family.

-State **“My father thinks abortion is murder.”** When prompted, you explain that you are not personally opposed to abortion, but are concerned about how pursuing abortion would impact your relationship to your family.

-When discussing adoption, say that you have a friend who is adopted and that she has a good life, but you think it would be really hard to do.

-When discussing parenting, say that you want to be a mom someday, but don’t know if you are ready to parent right now.

-If conversation is lagging and participant is not wrapping up, say, **“I just want to go home.”**

**Pregnancy Options Counseling OSCE**

**SP CHECKLIST-Kayla Johnson**

**SP: Student:**

**NOT**

**DONE DONE**

| 1. Assessed if patient aware that a pregnancy test has been ordered | **DONE** | **NOT**  **DONE** |
| --- | --- | --- |
| 1. Disclosed diagnosis of pregnancy in clear language, such as “You are pregnant” or “Your   pregnancy test is positive, which means that you are pregnant.”  **NOTE:** Saying “Your pregnancy test is positive” alone is not clear enough. | **DONE** | **NOT**  **DONE** |
| 1. When asked by patient if pregnancy test is accurate, affirmed that it is accurate. | **DONE** | **NOT**  **DONE** |
| 1. Allowed silence. Allowed patient time to react to diagnosis (at least 5 second pause). | **DONE** | **NOT**  **DONE** |
| 1. Inquired about and acknowledged patient’s feelings and reactions. | **DONE** | **NOT**  **DONE** |
| 1. Introduced pregnancy options, including parenting, adoption, and abortion. Need to discuss   **ALL THREE** options for credit.   1. **NOTE:** Need to mention all three of parenting, adoption, and abortion for credit. | **DONE** | **NOT**  **DONE** |
| 1. Avoided using terms like “keep the baby,” “give the baby up for adoption” or euphemisms for a   abortion such as “termination.” Preferred language is “parenting/become a parent,” “plan for  adoption,” and “abortion.” | **DONE** | **NOT**  **DONE** |
| 1. Assessed patient’s initial preferences about pregnancy options. Used a non-directive,   non-judgmental approach without leading in any particular direction. When prompted,  “What do you think I should do?” reflected decision-making back to patient. | **DONE** | **NOT**  **DONE** |
| 1. When asked about timeline for pregnancy decision-making, responded in a way that   balances allowing time for making a decision with information about time limitations for  abortion (i.e. “You don’t have to decide right now AND it’s good to make a decision sooner  rather than later, particularly if you’re considering abortion”) | **DONE** | **NOT**  **DONE** |
| 1. Asked patient if she would like to include a support person in the conversation. | **DONE** | **NOT**  **DONE** |
| 11. Demonstrated appropriate nonverbal behavior (eye contact, posture and position, facial expressions) | **DONE** | **NOT**  **DONE** |
| 12. Developed plan for next steps, including appropriate follow up and/or plans to disclose pregnancy to support person.  This could include discussing that the patient will follow up with someone to further discuss pregnancy options (i.e. responsible adult in her life, primary care provider, pregnancy options hotline counselor) and/or that she will contact particular pregnancy-related referrals if interested (i.e. prenatal provider, abortion provider, and/or adoption provider). Plan should be patient-centered and based in patient’s preferences and interests for next steps. | **DONE** | **NOT**  **DONE** |
| 13. Assessed safety (home and/or relationship safety). Must respond to prompt “My father is going to kill me” by probing for more information in some way (i.e. “Tell me more about that,” “What do you mean by that” “Do you feel safe at home” etc.). | **DONE** | **NOT**  **DONE** |
| 14. Offered resources about pregnancy options and/or emergency/crisis resources (either offered during the encounter or offered to return to bring resources back) | **DONE** | **NOT**  **DONE** |
| 15. Discussed prenatal health and safety (i.e. discussed at least one of the following: prenatal care, prenatal vitamins, avoidance of drugs/alcohol, review of prescription medications). | **DONE** | **NOT**  **DONE** |

16. Additional comments (please share what impacted you most during the encounter and why. Please share what exactly the learner did, and how it made you feel.):
