# Supplementary material for: Evaluation of a Pregnancy Options Counseling Curriculum for Pediatric Residents
Source: J Adolesc Health. Author manuscript; Available in PMC 2025 Mar 3. (PMC11875686; doi:10.1016/j.jadohealth.2024.11.003)
Supplement: Appendix 3 [file NIHMS2055353-supplement-Appendix_3.docx]

Appendix 3 Pregnancy Options Survey

Start of Block: Demographics

What are the last 4 digits of your phone number?

________________________________________________________________

What are the day and month of your birthday in 4 numbers? (I.e. June 22 = 0622)

________________________________________________________________

Have you ever received formal training before on pregnancy options counseling?

- Yes
- No
- Don't know

If yes, please specify what formal training you have previously received on pregnancy options counseling:

________________________________________________________________

End of Block: Demographics

Start of Block: Knowledge

What are the three options included as a part of options counseling?

- Abortion, D&C, contraception
- Termination, contraception, referral
- Abortion, delivery, termination
- Parenting, abortion, adoption
- Don’t know

In Pennsylvania, does a parent have to consent for a minor to have an abortion, unless a judge grants a bypass?

- Yes
- No
- Don't know

Which of the following are organizations to which you might refer a pregnant teenager in Pittsburgh, depending on their stage of decision-making?  Please select all that apply.

- All Options Talkline
- Teen Choices online workbook
- The Children's Home adoption agency
- University Women's Services
- Monongahela Sexual Health Center
- Allegheny Link
- Three Rivers Adoption Council

End of Block: Knowledge

Start of Block: Attitudes

How important is it for pediatric residents to be trained in options counseling?

- Very important
- Somewhat important
- A little important
- Not at all important
- Don't know

How much do you agree with the following statements?   When caring for a patient with an unintended pregnancy, I would be able to discuss the option of parenting

- Strongly agree
- Somewhat agree
- Somewhat disagree
- Strongly disagree
- Don't know

When caring for a patient with an unintended pregnancy, I would be able to discuss the option of adoption

- Strongly agree
- Somewhat agree
- Somewhat disagree
- Strongly disagree
- Don't know

When caring for a patient with an unintended pregnancy, I would be able to discuss the option of abortion

- Strongly agree
- Somewhat agree
- Somewhat disagree
- Strongly disagree
- Don't know

I feel that I am knowledgeable about pregnancy related resources

- Strongly agree
- Somewhat agree
- Somewhat disagree
- Strongly disagree
- Don't know

I feel that I could make a referral to a community resource for prenatal services

- Strongly agree
- Somewhat agree
- Somewhat disagree
- Strongly disagree
- Don't know

I feel that I could make a referral to a community resource for abortion services.

- Strongly agree
- Somewhat agree
- Somewhat disagree
- Strongly disagree
- Don't know

I feel that I could make a referral to a community resource for adoption services.

- Strongly agree
- Somewhat agree
- Somewhat disagree
- Strongly disagree
- Don't know

End of Block: Attitudes

Start of Block: Program Evaluation

How much do you agree with the following statements:   This session was valuable to my medical education

- Strongly agree
- Somewhat agree
- Somewhat disagree
- Strongly disagree
- Don't know

This session provided practical skills that will be useful to me during the rest of my medical training

- Strongly agree
- Somewhat agree
- Somewhat disagree
- Strongly disagree
- Don't know

The training was

- Far too short
- Somewhat too short
- Just the right amount of time
- Somewhat too long
- Far too long
- Don't know

What changes would you make if we were to conduct this training again?

________________________________________________________________

End of Block: Program Evaluation
